# Molecular characterization of Brazilian equid herpesvirus type 1 strains based on neuropathogenicity markers

**DOI:** 10.1590/S1517-838246220140096

**Published:** 2015-06-01

**Authors:** Enio Mori, Maria do Carmo C.S.H. Lara, Elenice M.S. Cunha, Eliana M.C. Villalobos, Claudia M.C. Mori, Rodrigo M. Soares, Paulo E. Brandão, Wilson R. Fernandes, Leonardo J. Richtzenhain

**Affiliations:** 1 Instituto Pasteur, São Paulo, SP, Brasil, Instituto Pasteur, São Paulo, SP, Brazil.; 2 Instituto Biológico, Instituto Biológico, São Paulo, SP, Brasil, Instituto Biológico, São Paulo, SP, Brazil.; 3 Universidade de São Paulo, Departamento de Patologia, Faculdade de Medicina Veterinária e Ciência Animal, Universidade de São Paulo, São Paulo, SP, Brasil, Departamento de Patologia, Faculdade de Medicina Veterinária e Ciência Animal, Universidade de São Paulo, São Paulo, SP, Brazil.; 4 Universidade de São Paulo, Departamento de Medicina Veterinária Preventiva e Saúde Animal, Faculdade de Medicina Veterinária e Zootecnia, Universidade de São Paulo, São Paulo, SP, Brasil, Departamento de Medicina Veterinária Preventiva e Saúde Animal, Faculdade de Medicina Veterinária e Zootecnia, Universidade de São Paulo, São Paulo, SP, Brazil.; 5 Universidade de São Paulo, Departamento de Clínica Médica, Faculdade de Medicina Veterinária e Ciência Animal, Universidade de São Paulo, São Paulo, SP, Brasil, Departamento de Clínica Médica, Faculdade de Medicina Veterinária e Ciência Animal, Universidade de São Paulo, São Paulo, SP, Brazil.

**Keywords:** equid herpesvirus type 1, equid Brazilian herpesvirus, *ICP4* gene (*ORF64*), glycoprotein D gene (*ORF72*), DNA polymerase gene (*ORF30*)

## Abstract

Partial nucleotide sequences of *ORF72* (glycoprotein D, *gD*), *ORF64* (infected cell protein 4, ICP4) and *ORF30* (DNA polymerase) genes were compared with corresponding sequences of EHV-1 reference strains to characterize the molecular variability of Brazilian strains. Virus isolation assays were applied to 74 samples including visceral tissue, total blood, cerebrospinal fluid (CSF) and nasal swabs of specimens from a total of 64 animals. Only one CSF sample (Iso07/05 strain) was positive by virus isolation in cell culture. EHV-1 Iso07/05 neurologic strain and two abortion visceral tissues samples (Iso11/06 and Iso33/06) were PCR-positive for ORF33 (glycoprotein B, *gB*) gene of EHV-1. A sequence analysis of the ORF72, *ORF64* and *ORF30* genes from three EHV-1 archival strains (A3/97, A4/72, A9/92) and three clinical samples (Iso07/05, Iso11/06 and Iso33/06) suggested that among Brazilian EHV-1 strains, the amplified region of the *gD* gene sequence is highly conserved. Additionally, the analysis of *ICP4* gene showed high nucleotide and amino acid identities when compared with genotype P strains, suggesting that the EHV-1 Brazilian strains belonged to the same group. All the EHV-1 Brazilian strains were classified as non-neuropathogenic variants (N752) based on the *ORF30* analysis. These findings indicate a high conservation of the gD-, *ICP4*- and *ORF30*-encoding sequences. Different pathotypes of the EHV-1 strain might share identical genes with no specific markers, and tissue tropism is not completely dependent on the *gD* envelope, immediate-early *ICP4* and DNA polymerase proteins.

Equid herpesvirus 1 (EHV-1) is the major cause of different clinical syndromes in horses, such as respiratory disease, abortion, neonatal deaths, and neurological disorders. It has been recognized as a cause of substantial financial losses to the horse industry throughout the world (Allen and Bryans, 1986; Gryspeerdt *et al.*, 2010).

Until recently, EHV-1 disease outbreaks usually manifested as abortions in late gestation; however, the frequency and severity of EHV-1 neurological diseases throughout North America and Europe have increased in recent years, and EHV-1 is now considered a potentially emerging disease of the horse by the US Department of Agriculture (Vandekerckhove *et al.*, 2010). Research studies have been performed suggesting that a molecular variation in the EHV-1 genome is playing a role in these changes in the disease behavior, which could indicate evolution of the viral agent (Pagamjav *et al.*, 2005; Nugent *et al.*, 2006). In Brazil, the first isolation of EHV-1 was recorded in 1966 from an equine-aborted fetus (Nilsson and Correa, 1966). After that, several isolates have been recovered, mainly from aborted fetuses; however, only recently a case report of EHV-1-related neurological signs in an adult mare was described (Lara *et al.*, 2008).

Restriction fragment length polymorphism (RFLP) analysis of whole DNA viral has been used to detect molecular variation among EHV-1 isolates. There are at least two electropherotype patterns of EHV-1 detected by restriction enzyme digestion designated EHV-1 P and EHV-1 B (Allen *et al.*, 1983). Based on previous studies, in the 3′-end and downstream of the open reading frame (ORF) 64 gene (infected cell protein 4 - *ICP4* gene), natural recombination between EHV-1 and EHV-4 by the exchange of homologous fragments could be associated with the major molecular differences between isolates EHV-1 P and EHV-1 B (Pagamjav *et al.*, 2005). The EHV-1 B genotype should be a result of this recombination between the progenitors of the EHV-1 P genotype and EHV-4. The *ICP4* is an important transcriptional activator, essential for progression beyond the immediate-early phase of infection, associated with lytic infection in HSV-1 (Pinnoji *et al.*, 2007). The *ICP4* product is involved in the regulation of gene expression and interaction with host factors, and this intertypic recombination could cause some alteration of EHV-1 virulence and neuropathogenicity in hamsters. The abortigenic genotype (EHV-1 B) may have originated from the neuropathogenic (EHV-1 P) after exchange of a fragment in the *ICP4* gene between EHV-1 and EHV-4 (Pagamjav *et al.*, 2005).

Glycoprotein D (*gD*) is responsible for virus entry and spread into a host cell, being major determinant of host cell tropism and may also be a factor involved in the neuropathogenicity of EHV-1 by modulating neurovirulence and neuroinvasion (Mettenleiter, 2003; Whalley *et al.*, 2007; Azab and Osterrieder, 2012).

EHV-1 molecular epidemiology research has identified a single nucleotide polymorphism (SNP) in the catalytic subunit (Pol) of the viral DNA polymerase (*ORF30*) gene, causing a substitution of asparagine (N) by aspartic acid (D) at amino acid position 752. This substitution showed a highly statistically significant (p < 0.0001) correlation with paralytic compared with non-paralytic disease outbreaks (Nugent *et al.*, 2006).

To the authors' knowledge, there have been few published articles on molecular variability of the EHV-1 Brazilian isolates. Despite the fact that ORF37 (similar to HSV-1 UL24) is considered a neuropathogenicity determinant of EHV-1 in the mouse encephalitis model (Kasem *et al.*, 2010), Carvalho *et al.* (2012) showed no molecular divergences on the partial sequencing of this region derived from two Brazilian EHV-1 isolates (A4/72 and A3/97) with high and low virulence in the mice model, respectively (Mori *et al.*, 2012).

The purpose of this study was to investigate some putative pathogenicity markers (*ICP4*, *gD* and viral DNA polymerase genes) in EHV-1 Brazilian strains to form a basis for comparison of partial nucleotide sequences with corresponding sequences of EHV-1 reference strains from DNA databases deposited in the GenBank (NCBI) to gather insights into the validity of such markers.

Three abortogenic (A4/72, A9/92 and A3/97) EHV-1 Brazilian archival strains, provided by the Biological Institute (Department of Agriculture, Sao Paulo State, Brazil), were recovered originally from organs (lungs, spleen and liver) of aborted fetuses. Viruses were propagated in Vero (CRL-1587, ATCC) and E-Derm (CCL-57, ATCC) cell lines and maintained in Eagle's minimal essential medium (EMEM) supplemented with 5% fetal bovine serum (FBS) at 37 °C in a humidified atmosphere of 5% CO_2_.

Seventy-four clinical specimens from horses (fragments of visceral tissues, total blood, CSF and nasal swab) were submitted for routine diagnostic tests to the Rabies and Viral Encephalitis subdivision of the Biological Institute (Department of Agriculture, Sao Paulo, Brazil) between 2005 and 2007 (Table 1). Sixty-four horses with an unknown vaccination history, and suggestive findings of EHV-1 infection [abortion (n = 25), neurological disease (n = 29), respiratory disease (n = 9) and perinatal disease (n = 1)] were sampled by private veterinarians from eight different Brazilian states: the Southeastern region [Sao Paulo state (n = 45), Minas Gerais state (n = 13), and Rio de Janeiro state (n = 1)]; the Midwestern region [Goias state (n = 1)]; South region [Parana state (n = 1), and Rio Grande do Sul (n = 1)]; and the Northeastern region [Rio Grande do Norte (n = 1), and Ceara (n = 1)].

**Table 1:** Animal groups (n = 64) and samples (n = 74) that were examined for the presence of EHV-1 by virus isolation and PCR during 2005 to 2007.

Group	Number of animals	Specimens					
		
		VT	BL	NT	CSF	NS	Pl
Abortion	25	24 (fetus)	-	1 (fetus)	-	-	1
Neurological disease	29	-	5	9	14	10	-
Respiratory disease	9	-	-	-	-	9	-
Perinatal disease	1	1 (foal)	-	-	-	-	-
Total	64	25	5	10	14	19	1

(-): Not collected; VT: visceral tissues (lung, liver and spleen); BL: total blood; NT: neuronal tissues; CSF: cerebrospinal fluid; NS: nasal swab; Pl: placenta.

Virus isolation (VI) was attempted with clinical samples (20% w/v brain or CSF) collected at necropsy and inoculated onto a monolayer of Vero (CRL-1587, ATCC) and E-derm (CCL-57, ATCC) cells. When these cells exhibited a cytopathic effect (CPE), the identification of isolates was performed according to previously published methods (Mori *et al.*, 2012).

DNA extraction of the EHV-1 Brazilian strains and specimens were conducted following a previously described method (Chomczynski, 1993). PCR screening tests (hemi-nested) were performed using primers that hybridize to highly conserved *gB* gene regions that differentiate between EHV-1 [P1 forward 5′-CTTGTGAGATCTAACC GCAC-3′/P2 outer reverse 5′-GGGTATAGAGCTTTC ATGGG-3′ and P1/P3 inner reverse 5′-GCGTTATAGC TATCACGTCC-3′] (Mori *et al.*, 2009) and EHV-4 [P4 forward 5′-CTGCTGTCATTATGCAGGGA-3′/P5 outer reverse 5′-CGTCTTCTCGAAGACGGGTA-3′ and P4/P6 inner reverse 5′-CGCTAGTGTCATCATCGTCG-3′] (Varrasso *et al.*, 2001). Next, three different sets of primers representing different regions of EHV-1 were used in positive amplification samples: *ICP4* gene [P7 forward 5′-ACGCCCCCTTCGTTCCTC-3′/P8 reverse 5′-CGCTCCACCTCGGTCCTG-3′] (Borchers *et al.*, 1998), *gD* gene [P9 forward 5′-ATGTCTACCTTCAAGCTT-3′/P10 reverse 5′-TTACGGAAGCTGGGTATA-3′] (Galosi *et al.*, 2001) and the DNA polymerase enzyme gene (*ORF30*) [P11 forward 5′-CCACAAACTTGATAAAC ACG-3′/P12 reverse 5′-GCGCTACTTCTGAAAACG-3′] (Nugent *et al.*, 2006). Amplification was performed in a reaction mixture of total volume 50 mL containing 0.5 mg of DNA sample, 0.5 mM of each primer, 0.2 mM of each dNTP mixture, 2.5 units of Platinum Taq DNA polymerase (Invitrogen Brasil Ltda, Sao Paulo, Brazil), 1 X PCR buffer (20 mM of Tris-HCl pH 8.4, 50 mM of KCl), 1.5 mM of MgCl and ultra-pure water QS. Amplification was carried out in a thermal cycler (Eppendorf Mastercycler Gradient PTC-200, Eppendorf AG, Hamburg, Germany) under the conditions reported in Table 2.

**Table 2:** Thermal cycling programs for PCR of EHV-4 gB primers and EHV-1 gB, gD, ICP4 and ORF30 primers.

Step		EHV-4	EHV-1			
			
		gB	gB	gD	ICP4	ORF30
1	Initial denaturation	95 °C (5-min)	94 °C (5-min)	94 °C (5-min)	96 °C (3-min)	94 °C (4-min)
2	Denaturation	95 °C (30-s)	94 °C (1-min)	94 °C (1-min)	94 °C (30-s)	94 °C (30-s)
3	Annealing	60 °C (30-s)	60 °C (1-min)	50 °C (1-min)	64 °C (30-s)	56 °C (1-min)
4	Extension	72 °C (1-min)	72 °C (1-min)	72 °C (90-s)	72 °C (1-min)	72 °C (2-min)
Number of cycles (step 2 to 4)		35	35	25	35	35
5	Final extension	72 °C (5-min)	72 °C (7-min)	72 °C (6-min)	72 °C (6-min)	72 °C (10-min)

A commercial kit (GFX PCR DNA and Gel Band Purification Kit, GE Healthcare, Uppsala, Sweden) was used for the purification of amplified DNA fragments. Then, bidirectional cycle sequencing was performed using the dideoxynucleotide chain-termination method (Big Dye Terminator v.3.1 Cycle Sequencing Kit, Applied Biosystems, Foster City, California, USA) according to the manufacturer's instructions. Sequence reaction products were analyzed on an automatic DNA sequencer (ABI Prism 377 Genetic Analyzer, Applied Biosystems Foster City, California, USA). The sequence quality analysis was examined by the *Phred* program (http://asparagin.cenargen.embrapa.br/phph/). The lower threshold of acceptability for the generation of consensus sequences was set at a *Phred* score of 20 for each base. Next, the final sequences were assembled using the contig assembly program (CAP) of the software BioEdit v.7.0.9.0 (Hall, 1999). Sequences of each EHV-1 strain and homologous sequences retrieved from GenBank were aligned by the ClustalW method using Bioedit v.7.0.9.0 (Hall, 1999). Nucleotide and amino acid identities of the translated sequences were calculated using Bioedit v.7.0.9.0 (Hall, 1999).

Phylogenetic tree using the sequences from the *ORF64* gene region were carried out using the neighbor-joining (NJ) algorithm and the maximum composite likelihood (MCL) evolutionary model with software Mega version 6.0.6 (Tamura *et al.*, 2013). The reliability of the NJ phylogenetic trees was evaluated by analyzing 1,000 bootstrap repetitions, and the virus HHV-3 strain Dumas (accession number NC001348) was used as an outgroup. The genomic partial sequences of the EHV-1 strains [Ab4p (accession number AY665713), KyD (accession number AB279610), V592 (accession number AY464052), RacL11 (accession number AB279607), KyA (accession number M629230), Ab1 (accession number M60946), HVS25A (accession number M59773), 98c12 (accession number AB183143), 97c5 (accession number AB183141), 97c7 (accession number AB182650) and 97c9 (accession number AB183142)], the EHV-9 strain P19 (accession number NC011644) and the EHV-4 strain NS80567 (accession number AF030027) were obtained from DNA databases that had been deposited in the GenBank (NCBI) and used for comparison purposes.

Virus isolation assays were applied to 74 samples including visceral tissue, total blood, cerebrospinal fluid (CSF) and nasal swabs of farms specimens from a total of 64 animals (Table 1). Only one cerebrospinal fluid (CSF) sample (namely strain Iso07/05) of a mare with a neurological disorder from a riding school in Ribeirao Pires County (Sao Paulo State, Southeastern Brazil) caused herpesvirus CPE after the first passage in ED cells (Lara *et al.*, 2008). EHV-1 Iso07/05 neurologic strain and two abortion visceral tissue (VT) samples (Iso11/06 and Iso33/06) were positive for EHV-1 PCR with *gB* primers. Iso11/06 and Iso33/06 samples were originated from Belo Horizonte County (Minas Gerais State, Southeastern Brazil) and Pirassununga County (Sao Paulo State, Southeastern Brazil), respectively.

The use of *gD*, *ICP4* and *ORF30* as primers showed that the Brazilian archival EHV-1 strains (A4/72 and A3/97) were positive by PCR, whereas A9/92 strain was positive only for *ICP4* primers. *ICP4*, *gD* and *ORF30* regions of the EHV-1 Brazilian sequences were deposited in GenBank (accession numbers EU094656, EU094657, EU088186, EU088187, EU410444, EU410445 and EU094655). EHV-1 strains (Iso07/05, Iso11/06 and Iso33/06) were PCR-positive using *gD*, *ICP4* and *ORF30* gene primers. The positive PCR amplicons *gD*, *ICP4* and *ORF30* genes were partially sequenced (GenBank accession numbers EU052212, EU169121, EU410443, JN390439, EU825794, EU857541, JN390440, EU825795, and FJ755482). The *gD* gene amplicon lengths were 935 nt and encoded 310 amino acids. At the nucleotide level of the *gD* gene, the Brazilian EHV-1 isolates and clinical samples showed 100% identity among them. Comparing Brazilian EHV-1 isolates and clinical specimens with those deposited in GenBank, it was observed that nucleotide and predicted amino acid sequences of the *gD* exhibited high identities (99.6–100% and 99–100%, respectively), differing in few nucleotides and resulting in low rates of amino acid change (Supplementary Figure S3). The results suggested that among Brazilian EHV-1 strains, the *gD* gene is highly conserved, thus supporting the use of vaccines that contain DNA or subunits related to this region.

Although there are dramatic differences in the virulence and tissue tropism between A4/72 and A3/97 after intranasal inoculation with the same viruses (Mori *et al.*, 2012), the highly conserved region of *gD* do not explain the pathogenetic differences of the EHV-1 Brazilian isolates. However, a strain with different pathogenicity in mice might have identical *gD*s, a fact not reported previously.

In contrast to the attenuated EHV-1 strains (KyA, KyD and RacL11) used as vaccine, drastic mutations in *gD* sequence, such as deletion, inversion, and insertion, were not found in the strains here analyzed (Supplementary Figure S1). A possible explanation for DNA mutations in KyA, KyD and RacL11 strains may be due to serial passage in hamsters and culture cells (Molinkova *et al.*, 2004; Ghanem *et al.*, 2007).

The nucleotide sequence of the *ICP4* region was 309nt long and encoded 102 amino acids. At both the nucleotide and the amino acid levels of the *ICP4*, the Brazilian EHV-1 isolates and clinical samples showed 100% identity among them. Comparison of the *ICP4* nucleotide and amino acid sequences obtained from Brazilian EHV-1 isolates and clinical specimens with those from the EHV-1 genotype P strains (Ab4p and V592) exhibited 100% identity, suggesting that these viruses belonged to the same group (Pagamjav *et al.*, 2005). The genealogic tree for the *ORF64* gene constructed with the sequences analyzed in this study clustered in only one group named genotype P (Figure 1).

**Figure 1:**
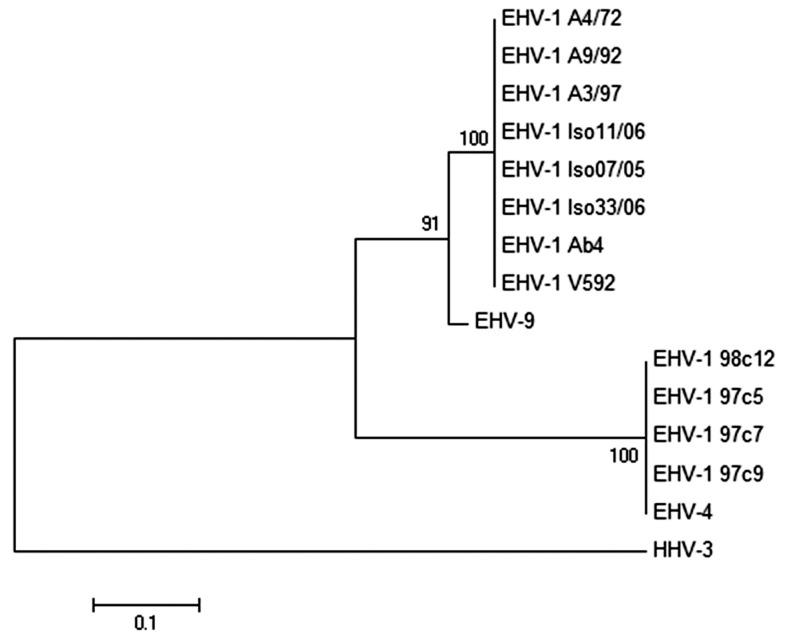
Neighbor-joining genealogic tree using partial *ORF64* nucleotide sequence (nt) from EHV-1 Brazilian strains (A4/72, A9/92, A3/97, Iso11/06, Iso07/05 and Iso33/06), GenBank reference EHV-1 strains (Ab4, V592, 98c12, 97c5, 97c7 and 97c9) and GenBank reference alphaherpesviruses strains (EHV-9 strain P19, EHV-4 strain NS80567 and HHV-3 strain Dumas). The numbers at each node are bootstrap values. EHV-1 genotype P strains [A4/72 (accession number EU094657), A9/92 (accession number EU094655), A3/97 (accession number EU094656), Iso11/06 (accession number EU825794), Iso07/05 (accession number EU169121), Iso33/06 (accession number EU825795), Ab4p (accession number AY665713) and V592 (accession number AY464052)], EHV-1 genotype B strains [98c12 (accession number AB183143), 97c5 (accession number AB183141), 97c7 (accession number AB182650) and 97c9 (accession number AB183142)], EHV-9 strain P19 (accession number NC011644), EHV-4 strain NS80567 (accession number AF030027) and HHV-3 strain Dumas (accession number NC001348).

On the other hand, the nucleotide sequences of the *ICP4* in Brazilian EHV-1 strains exhibited 69.6% nucleotide identity with EHV-1 genotype B strains (97c5, 97c7, 97c9 and 98c12) and EHV-4 (strain NS80567). In addition, the Brazilian strains exhibited 49% amino acid identity with EHV-1 genotype B strains and EHV-4.

Pagamjav *et al.* (2005) suggested that the intertypic recombination in the *ICP4* gene could cause an alteration in EHV-1 virulence and neuropathogenicity in the hamster model. The EHV-1 P strains were correlated with neuropathogenic behavior in hamster model. However, as occurred with the *gD* gene region, the involvement of this gene in neuropathogenicity in mice could not be confirmed based on the results of the *ICP4* nucleotide sequencing from the EHV-1 Brazilian isolates (Mori *et al.*, 2012).

The *ORF30* nucleotide sequences were 426nt long and encoded 141 amino acids. The DNA polymerase gene region of the EHV-1 Brazilian isolates and clinical specimens showed 100% nucleotide identity with the non-neuropathogenic variant (N752) EHV-1 strain V592. Nucleotide and amino acid identity among the Brazilian strains and the neuropathogenic variant (D752) was 99.7% and 99.2%, respectively.

Although strain Iso07/05 was recovered from CSF and classified as a neurotropic isolate, all the EHV-1 Brazilian strains were classified as non-neuropathogenic (N752) in the catalytic subunit (Pol) of the viral DNA polymerase gene (Nugent *et al.*, 2006). Nugent *et al.* (2006) found that approximately 15% of isolates from cases of EHV-1 neurological disease did not contain the mutation in this gene.

The A9/92 strain could have differences in its DNA composition in comparison with other EHV-1 strains, which could explain the different ways of spreading and the neurological signs A9/92 strain causes in mice model (Mori *et al.*, 2012).

This is one of the first molecular epidemiological investigations into EHV-1 Brazilian isolates, and it does not reveal any molecular variation in the *ICP4*, *gD* and viral DNA polymerase gene regions among these strains. These results suggest that other factors, such as immune response, could be involved in the neuropathogenicity of EHV-1 in the mouse models (Mori *et al.*, 2012). In conclusion, different pathotypes of EHV-1 might share identical genes with no specific markers, and tissue tropism is not completely dependent on the *gD* envelope, immediate-early *ICP4* and DNA polymerase proteins. Further studies of other potential neurovirulence markers are required to clarify the relationship between molecular variation and enhanced virulence in the mouse model, which may help elucidate the neuropathogenicity of particular strains of EHV-1.

## Supplementaty Material

Figure S1: Amino acid sequence alignment among EHV-1 Brazilian isolates (A4/72, A3/97, ISO07/05, ISO11/06 and ISO33/06) and Genbank reference EHV-1 strains (Ab4, KyD, V592, RacL11, HVS25A, KyA and Ab1) gD sequences.
