# Supplementary material for: Molecular characterization of Brazilian equid herpesvirus type 1 strains based on neuropathogenicity markers
Source: Braz J Microbiol. 2015 Jun 1;46(2):565–70. doi: 10.1590/S1517-838246220140096 (PMC4507552; doi:10.1590/S1517-838246220140096)
Supplement: Supplementary file 1 [file 1517-8382-bjm-46-2-565-suppl01.pdf]

Supplementary Figure S1 Amino acid sequence alignment among EHV-1 Brazilian isolates (A4/72, A3/97, ISO07/05, ISO11/06 and ISO33/06) and Genbank reference EHV-1 strains (Ab4, KyD, V592, RacL11, HVS25A, KyA and Ab1) gD sequences. Numbers correspond to amino acid positions.Identical residues are indicated by dots.The alignment was obtained using ClustalWmethod.

```
                    10        20        30        40        50        60        70        80        90       100
           ....|....|....|....|....|....|....|....|....|....|....|....|....|....|....|....|....|....|....|....|
Ab4        PPPRYNYTILTRYNATALASPFINDQVKNVDLRIVTATRPCEMIALIAKTNIDSILKELAAAQKTYSARLTWFKIMPTCATPIHDVSYMKCNPKLSFAMC
KyD        ....................................................................................................
V592       ....................................................................................................
RacL11     ....................................................................................................
HVS25A     ....................................................................................................
KyA        ....................................................................................................
Ab1        ....................................................................................................
A4/72      ....................................................................................................
A3/97      ....................................................................................................
ISO07/05   ....................................................................................................
ISO11/06   ....................................................................................................
ISO33/06   ....................................................................................................

                   110       120       130       140       150       160       170       180       190       200
           ....|....|....|....|....|....|....|....|....|....|....|....|....|....|....|....|....|....|....|....|
Ab4        DERSDILWQASLITMAAETDDELGLVLAAPAHSASGLYRRVIEIDGRRIYTDFSVTIPSERCPIAFEQNFGNPDRCKTPEQYSRGEVFTRRFLGEFNFPQ
KyD        ....................................................................................................
V592       ....................................................................................................
RacL11     ....................................................................................................
HVS25A     ....................................................................................................
KyA        ..............................................................L.....................................
Ab1        ..............................................Q.....................................................
A4/72      ....................................................................................................
A3/97      ....................................................................................................
ISO07/05   ....................................................................................................
ISO11/06   ....................................................................................................
ISO33/06   ....................................................................................................

                   210       220       230       240       250       260       270       280       290       300
           ....|....|....|....|....|....|....|....|....|....|....|....|....|....|....|....|....|....|....|....|
Ab4        GEHMTWLKFWFVYDGGNLPVQFYEAQAFARPVPPDNHPGFDSVESEITQNKTDPKPGQADPKPNQPFKWPSIKHLAPRLDEVDEVIEPVTKPPKTSKSNS
KyD        ....................................................................................................
V592       ....................................................................................................
RacL11     ....................................................................................................
HVS25A     ....................................................................................................
KyA        .V.....................................................................V.............................
Ab1        ....................................................................................................
A4/72      ....................................................................................................
A3/97      ....................................................................................................
ISO07/05   ....................................................................................................
ISO11/06   ....................................................................................................
ISO33/06   ....................................................................................................

                   310
           ....|....|
Ab4        TFVGISVGLG
KyD        ..........
V592       ..........
RacL11     ..........
HVS25A     ..........
KyA        ..........
Ab1        ..........
A4/72      ..........
A3/97      ..........
ISO07/05   ..........
ISO11/06   ..........
ISO33/06   ..........
```

Ab4p (accession number AY665713), KyD (accession number AB279610), V592 (accession number AY464052), RacL11 (accession number AB279607), HVS25A (accession number M59773), KyA (accession number M629230), Ab1 (accession number M60946), A4/72 (accession number EU088187), ISO07/05 (accession number EU052212), A3/97 (accession number EU088186), ISO 11/06 (accession number JN390439) and ISO33/06 (accession number JN390440).
